# A Rare Presentation of Non-Hodgkin’s Lymphoma Presenting With Bleeding Esophageal Varices Secondary to a Malignant Portal Vein Tumor Thrombosis

**DOI:** 10.7759/cureus.9162

**Published:** 2020-07-13

**Authors:** Eric M Sieloff, Alpana Garg, Sachin Goyal, Anjali Alangaden, Kirthi K Lilley

**Affiliations:** 1 Internal Medicine, Western Michigan University Homer Stryker M.D. School of Medicine, Kalamazoo, USA; 2 Internal Medicine, Wayne State University Detroit Medical Center, Detroit, USA; 3 Gastroenterology, Wayne State University, Detroit, USA; 4 Medicine, Wayne State University Detroit Medical Center, Detroit, USA; 5 Gastroenterology, Wayne State University School of Medicine, Detroit, USA; 6 Gastroenterology, Detroit Veterans Affairs Medical Center, Detroit, USA

**Keywords:** portal vein, deep vein thrombosis, non-hodgkin lymphoma, diffuse large b-cell lymphoma, portal hypertension, esophageal varices

## Abstract

A 60-year-old woman presented with six months of abdominal pain, weight loss and diarrhea for which she underwent bidirectional endoscopies that were unremarkable. Over the next two weeks, she developed non-cirrhotic portal hypertension and presented with esophageal variceal bleeding. A diffuse large B-cell lymphoma encircling her celiac axis with a tumor thrombosis of the superior mesenteric, splenic and portal veins was found to be the cause of her portal hypertension. An esophagogastroduodenoscopy (EGD) was performed to control her variceal bleeding. She was started on R-CHOP (rituximab, cyclophosphamide, doxorubicin, vincristine and prednisone) chemotherapy and after three cycles her symptoms have subsided, and a CT scan has shown shrinking mesenteric lymphadenopathy.

## Introduction

A portal vein thrombosis (PVT) is when a thrombus occludes the portal vein partially, or completely, though such phenomenon can extend to include both the splenic and mesenteric veins. Untreated, the consequences of a PVT can be devastating and include bowel ischemia and portal hypertension [1]. The etiology of a PVT is often multifactorial and can include liver cirrhosis, inherited or acquired thrombophilias, intra-abdominal inflammatory processes and malignancies in terms of associated prothrombotic states, vascular invasion and extravascular compression [2]. Malignant causes of PVT, or portal vein tumor thrombosis (PVTT), are often intra-abdominal solid tumors [3]. We present a rare cause of PVTT secondary to localized non-Hodgkin's lymphoma (NHL) encircling the celiac axis causing extrinsic compression and intravascular invasion, associated with non-cirrhotic portal hypertension and bleeding esophageal varices for which only a handful of cases have been reported in medical literature.

## Case presentation

A 60-year-old Caucasian woman with no significant past medical history presented to our gastroenterology ambulatory clinic with complaints of chronic lower abdominal pain and diarrhea for the past six months. She had no personal history of malignancy, and her family history was significant only for a brother with prostate cancer. She described her abdominal pain as crampy in nature, exacerbated by food, radiating to the left side and relieved by defecation. She had been having significant diarrhea up to 10-20 episodes daily which were non-bloody and watery. In an attempt to control her bowel movements, she had been taking up to four Imodium tablets daily with some improvement. She also noted a weight loss of 30-35 pounds in the past three months which she attributed to early satiety and the fact that she had been self-restricting her diet to include liquids only. She reported subjective fevers in the evenings and having night sweats over the past six months but denied any nausea or hematochezia.

Laboratory values were unremarkable without any new electrolyte imbalances, leukocytosis and thrombocytopenia, and her hemoglobin was at her baseline of 10 g/dL. Her carcinoma embryonic antigen (CEA) and carbohydrate antigen (CA 19-9) values were normal. She underwent esophagogastroduodenoscopy (EGD) and colonoscopy which were normal, along with normal duodenal and gastric biopsies. Plans were made to proceed with abdominal imaging and return to clinic afterwards.

**Figure 1:**
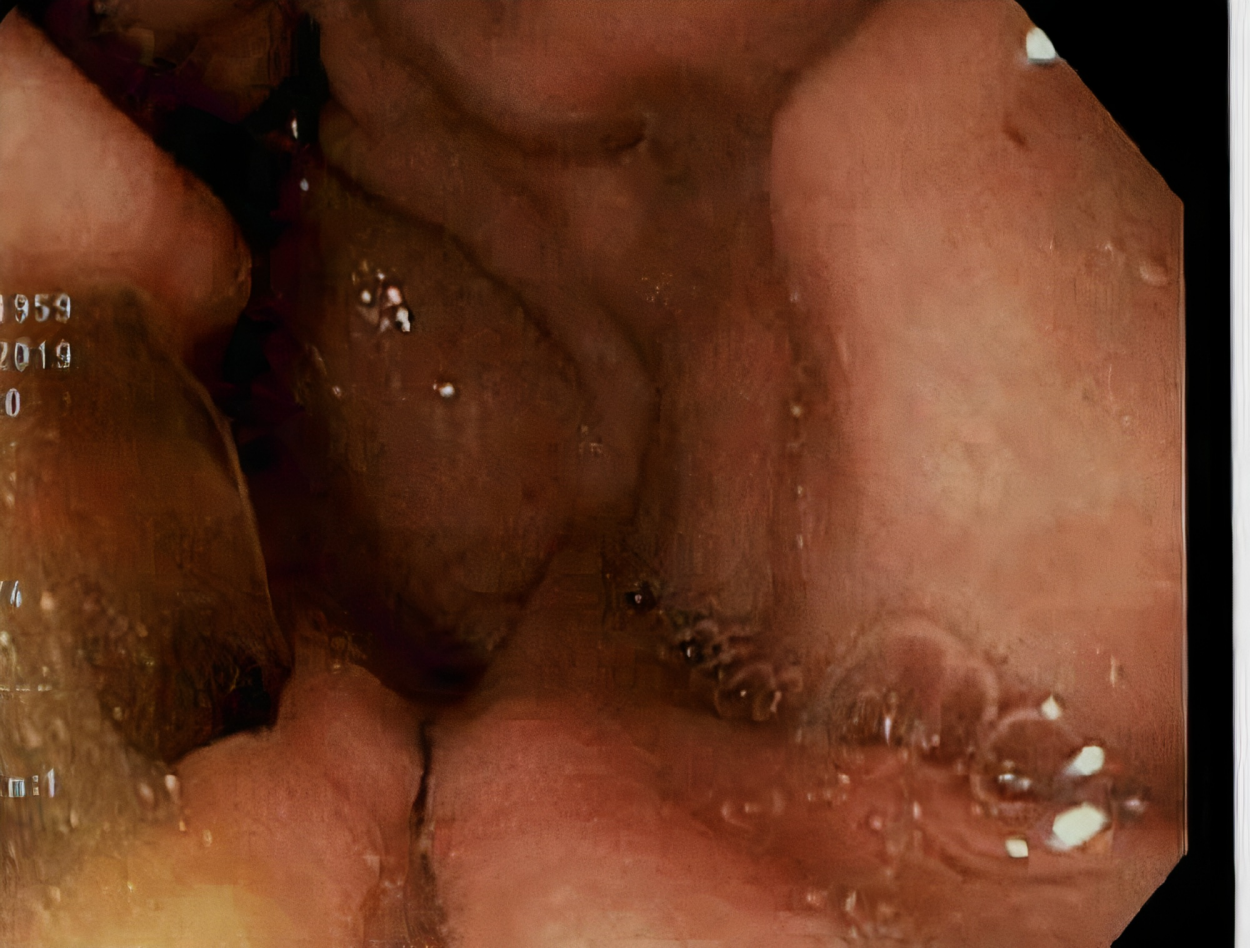
Esophagogastroduodenoscopy showing evidence of newly developed grade II esophageal varices.

While waiting for further investigation, approximately two weeks later, she presented to our hospital with complaints of two days of melena and her hemoglobin was found to be 7.6 g/dL while all her other labs remained unremarkable. On physical exam, her abdomen had become diffusely tender to deep palpation and a firm mobile mass could be felt superior to the umbilical region. There was no obvious cervical or axillary lymphadenopathy. An EGD was repeated and revealed grade II lower esophageal varices with red wale signs, suggestive of recent bleeding, and endoscopic variceal band ligation was done (Figure 1).

**Figure 2:**
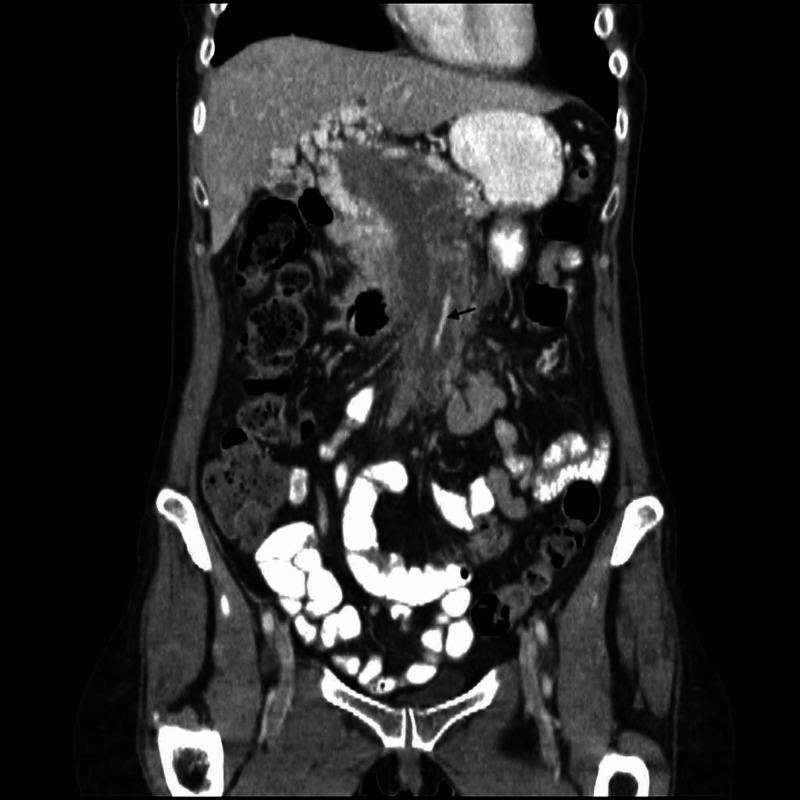
Coronal plane abdominal contrast-enhanced CT showing a large (7.2 cm x 2.5 cm) heterogeneous soft tissue mass within the root of the mesentery surrounding the superior mesenteric artery (black arrow).

**Figure 3:**
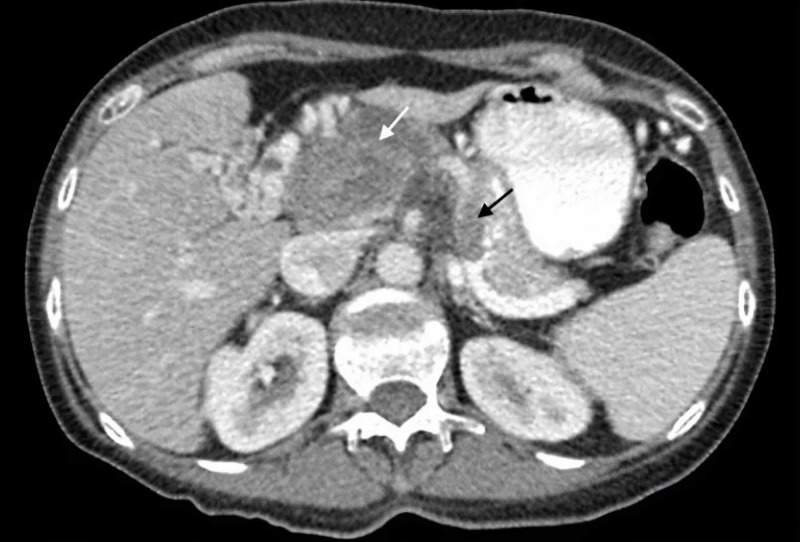
Axial plane abdominal contrast-enhanced CT showing a mass lesion (white arrow) adjacent to liver and aorta, and a low attenuation filling defect in the lumen of splenic vein (black arrow).

A CT scan of the abdomen and pelvis was performed and showed a 7.2 cm x 2.5 cm heterogeneously enhancing soft tissue mesenteric mass with necrosis surrounding the celiac trunk and the superior mesenteric artery. It also revealed an additional extensive tumor thrombus extending from the mesenteric mass to involve the superior mesenteric, splenic and portal veins (Figures 2, 3). 

**Figure 4:**
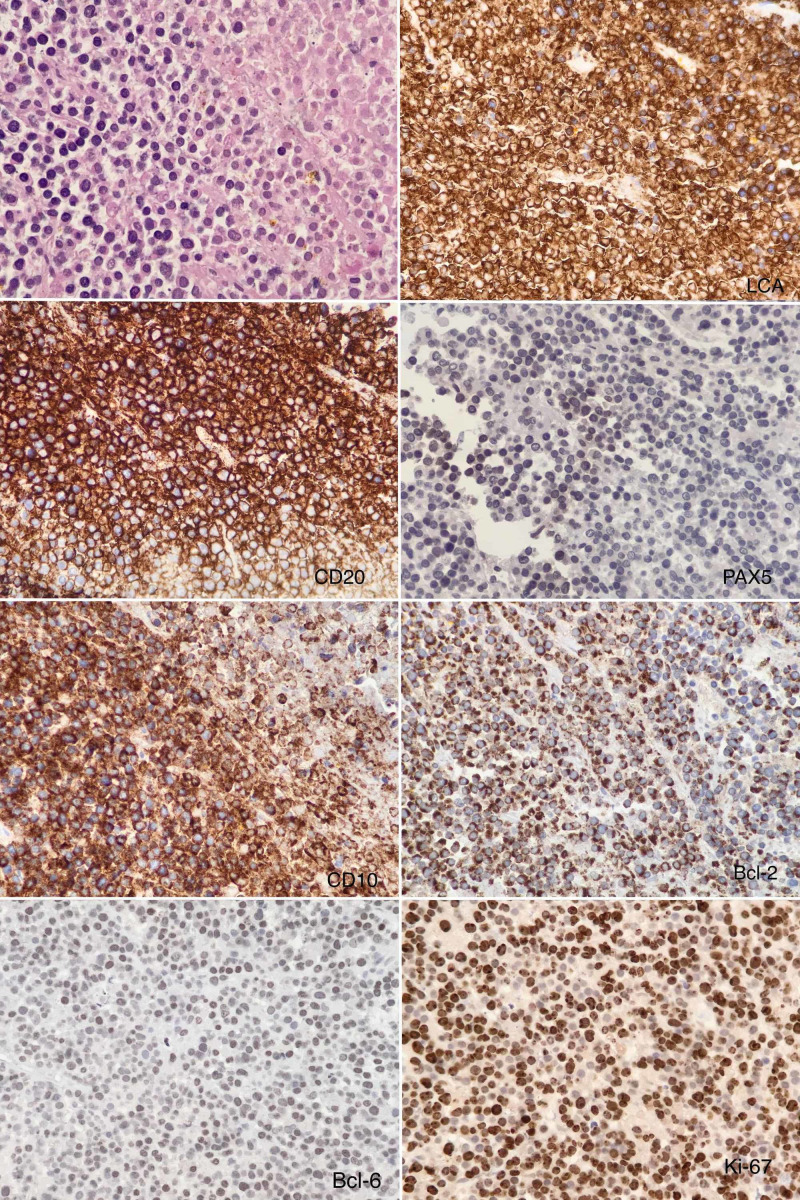
Histologic examination of diffuse large B-cell lymphoma non-Hodkgin’s lymphoma with hematoxylin and eosin staining (×400 magnification) followed by LCA, CD20, PAX-5, CD10, BCL-2, BCL-6 and Ki-67 staining.

She underwent a CT-guided biopsy of this mass which showed high-grade diffuse large B-cell lymphoma (DLBCL) NHL with a Ki-67 of 80%. Morphologically, no granulomas or Reed-Sternberg cells were seen and further immunostaining performed showed positivity for LCA, CD20, PAX-5 (weak and focal), CD10 (diffuse), BCL-2 (diffuse) and BCL-6 (about 30%), while being negative for CD3, CD5, CD20, CD23, cyclin D1, MUM1, c-MYC, AE1/AE3, CAM 5.2, CK7 and CK20, suggesting NHL DLBCL (Figure 4).

**Figure 5:**
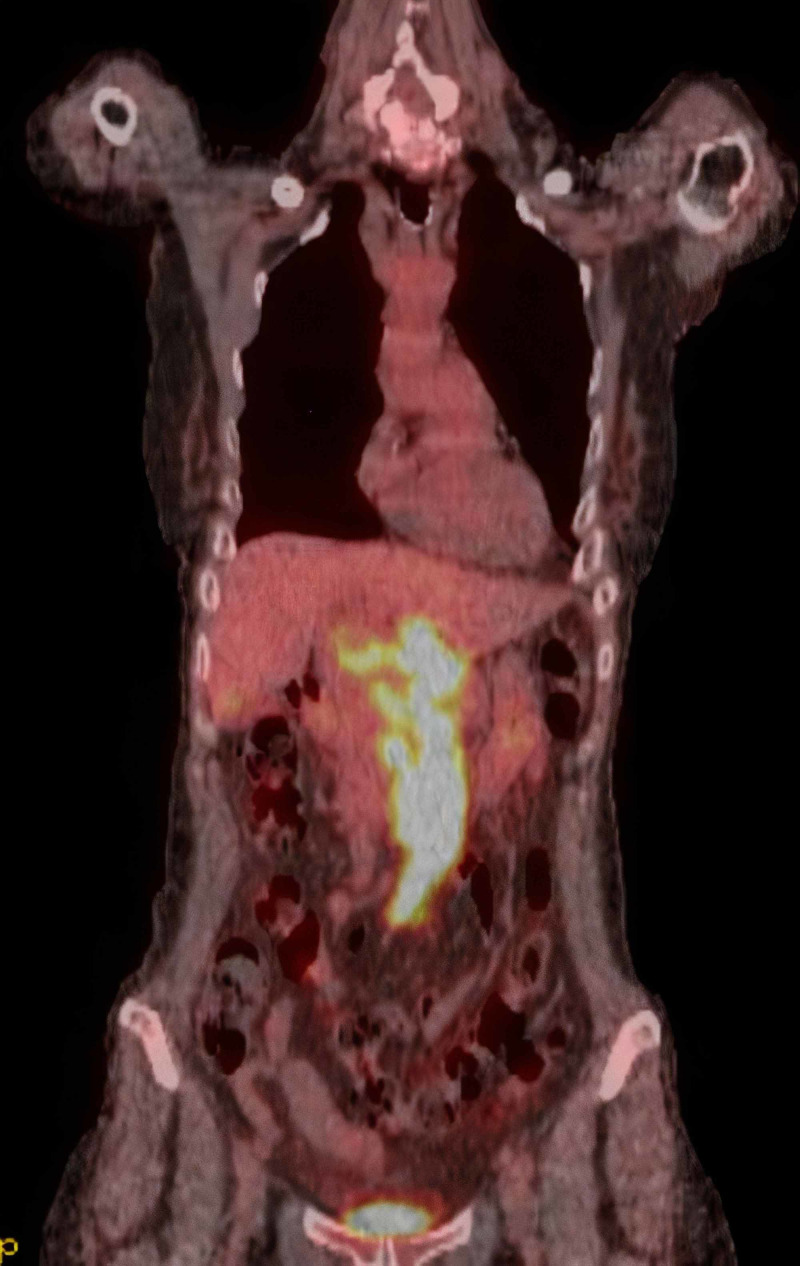
Positron emission tomography/CT scan showing a localized 18 F-fluorodeoxyglucose (FDG)-avid mass surrounding the portal vein.

A positron emission tomography (PET)-CT was performed and demonstrated an 18 F-fluorodeoxyglucose (FDG) avid mass with surrounding mesenteric lymphadenopathy and no evidence of disease elsewhere (Figure 5).

Our patient was started on R-CHOP (rituximab, cyclophosphamide, doxorubicin, vincristine and prednisone) chemotherapy immediately. After three cycles of her chemotherapy regimen, her symptoms have subsided without any further evidence of gastrointestinal bleeding. A repeat CT scan showed that her mesenteric lymphadenopathy has begun to shrink, though the PVTT remains unchanged. She has been maintained on propanalol for secondary prophylaxis of esophageal varices and has experienced no further gastrointestinal bleeding. She continues to follow with hematology oncology for the management of NHL. She is also scheduled for a repeat EGD to assess for any remaining varices needing further obliteration.

## Discussion

This case describes an unusual etiology of esophageal variceal bleeding secondary to non-cirrhotic portal hypertension from an extensive PVTT caused by an NHL malignancy surrounding the celiac trunk. Current medical literature yields only seven previously reported cases of NHL-associated PVTT, of which four were specifically DLBCL [4-10]. This makes our case the eighth reported presentation of NHL-associated PVTT in medical literature, and the fifth specifically associated with DLBCL. Many of these documented cases of NHL PVTT have had great success with CHOP chemotherapy, alone, resulting in PVTT resolution [4,5,7,8]. One of the earliest cases of NHL PVTT from 1996 documented by Irie and colleagues reported no specific patient outcomes [9]. Ten years later, a case of DLBCL-associated NHL PVTT reported by Kanemura and colleagues had a near-complete resolution of the PVTT burden with CHOP chemotherapy associated with esophageal variceal obliteration signifying the resolution of non-cirrhotic portal hypertension [6]. Lastly, a case of marginal zone B-cell lymphoma PVTT with a concurrent myeloproliferative neoplasm was associated with full recanalization of the portal vein with CHOP therapy [10]. 

Localized NHL, despite its various presentations, is an exceedingly uncommon cause of a PVTT as such pathology is more often seen secondary to pancreatic adenocarcinoma, hepatocellular carcinoma, renal cell carcinoma and cholangiocarcinoma [11]. A localized malignancy causing PVTT can appear as a heterogeneous mass on Doppler ultrasound invading the portal vein, while CT can be used to assess the extent of vascular involvement. It is important to recognize that in the event of NHL-associated tumor thrombosis, esophageal varices can form as the result of a PVTT, while gastric varices can be the result of a splenic vein thrombosis [12]. Regardless, treatment of a PVTT is crucial: a thrombus extension into the superior mesenteric vein can result in intestinal ischemia with potential perforation, and a chronic tumor thrombus burden can result in portal hypertension with portosystemic shunts and bleeding varices, as seen in our case [1]. Endoscopic variceal ligation can be used for immediate treatment of bleeding esophageal varices, while non-selective beta-blockers can be used as prophylaxis [13]. In cases of NHL tumor thrombosis from extrinsic vascular compression and intravascular invasion, thrombus resolution is often seen with CHOP chemotherapy [4,5,14-16]. Currently, our patient’s symptoms have subsided and she is scheduled for additional chemotherapy treatments with imaging studies to assess for PVTT resolution, and an EGD to investigate for any remaining esophageal varices.

## Conclusions

We present a case of a patient with a malignant PVTT with associated non-cirrhotic portal hypertension and esophageal variceal bleeding secondary to localized NHL around the celiac axis which is exceedingly rare. As such thrombosis was suspected to be primarily from extrinsic tumor compression and intravascular invasion, she has been started on R-CHOP chemotherapy. After three cycles of treatment, her symptoms have subsided entirely, and a repeat CT scan shows evidence of shrinking mesenteric lymphadenopathy though the PVTT remains in place. With further treatments, we hope to see further remission of her NHL and full resolution of her PVTT. Although the etiologies of a PVT can be extensive, and often multifactorial, localized NHL can be considered as part of the differential diagnosis.
